# Deficiency of Nrf2 exacerbates white matter damage and microglia/macrophage levels in a mouse model of vascular cognitive impairment

**DOI:** 10.1186/s12974-020-02038-2

**Published:** 2020-12-01

**Authors:** Emma Sigfridsson, Martina Marangoni, Giles E. Hardingham, Karen Horsburgh, Jill H. Fowler

**Affiliations:** 1 Centre for Discovery Brain Sciences, University of Edinburgh, Chancellor’s Building, 49 Little France Crescent, Edinburgh, EH16 4SB UK; 2 Present address: Department of Health Sciences, University of Florence, Florence, Italy; 3 The UK Dementia Research Institute, University of Edinburgh, Edinburgh, UK

**Keywords:** Chronic cerebral hypoperfusion, Oxidative stress, Inflammation, White matter, Nrf2, Microglia, Astrocytes, C4, C1q, Ccl3

## Abstract

**Background:**

Chronic cerebral hypoperfusion causes damage to the brain’s white matter underpinning vascular cognitive impairment. Inflammation and oxidative stress have been proposed as key pathophysiological mechanisms of which the transcription factor Nrf2 is a master regulator. We hypothesised that white matter pathology, microgliosis, blood-brain barrier breakdown and behavioural deficits induced by chronic hypoperfusion would be exacerbated in mice deficient in the transcription factor Nrf2.

**Methods:**

Mice deficient in Nrf2 (male heterozygote or homozygous for Nrf2 knockout) or wild-type littermates on a C57Bl6/J background underwent bilateral carotid artery stenosis (BCAS) to induce chronic cerebral hypoperfusion or sham surgery and survived for a further 6 weeks. White matter pathology was assessed with MAG immunohistochemistry as a marker of altered axon-glial integrity; alterations to astrocytes and microglia/macrophages were assessed with GFAP and Iba1 immunohistochemistry, and blood-brain barrier breakdown was assessed with IgG immunohistochemistry. Behavioural alterations were assessed using 8-arm radial arm maze, and alterations to Nrf2-related and inflammatory-related genes were assessed with qRT-PCR.

**Results:**

Chronic cerebral hypoperfusion induced white matter pathology, elevated microglial/macrophage levels and blood-brain barrier breakdown in white matter tracts that were increased in Nrf2^+/−^ mice and further exacerbated by the complete absence of Nrf2. Chronic hypoperfusion induced white matter astrogliosis and induced an impairment in behaviour assessed with radial arm maze; however, these measures were not affected by Nrf2 deficiency. Although Nrf2-related antioxidant gene expression was not altered by chronic cerebral hypoperfusion, there was evidence for elevated pro-inflammatory related gene expression following chronic hypoperfusion that was not affected by Nrf2 deficiency.

**Conclusions:**

The results demonstrate that the absence of Nrf2 exacerbates white matter pathology and microgliosis following cerebral hypoperfusion but does not affect behavioural impairment.

## Background

Sustained cerebral hypoperfusion is one of the pathophysiological mechanisms contributing to cognitive decline in ageing, vascular cognitive impairment (VCI) and Alzheimer’s disease (AD) by causing damage to the brain’s white matter [1–3]. White matter hyperintensities (WMH) are frequently detected in ageing, AD and VCI, and MRI and neuropathological investigation suggest they can be attributed to cerebral hypoperfusion [3, 4]. Furthermore, the burden of WMH has been correlated with a decline in global cognitive performance, executive function and processing speed [5, 6]. The severity of hypoperfusion predicts conversion from mild cognitive impairment to dementia [7, 8].

Animal models of chronic cerebral hypoperfusion have provided evidence for a causal link between cerebral hypoperfusion, white matter pathology and cognitive deficits (reviewed in [1]). We demonstrated that bilateral carotid artery stenosis (BCAS) in mice causes chronic cerebral hypoperfusion [9], white matter alterations detected using diffusion-tensor MRI [10] and impaired axon-glial integrity [11, 12]. Furthermore, we showed that the white matter disruption caused by BCAS induces a selective deficit in spatial working memory [11], reminiscent of the disrupted frontal cortical circuitry found in VCI, that progresses to encompass spatial reference memory deficits in the longer term response [13].

An elevated inflammatory response and oxidative stress are implicated in the pathogenesis of white matter damage following cerebral hypoperfusion. Elevated levels of microglia/macrophages parallel white matter damage after chronic cerebral hypoperfusion and correlate with impaired white matter function and cognitive deficits [9, 11, 14–16]. At a mechanistic level, activated microglia may damage white matter after chronic hypoperfusion by sustained elevation of pro-inflammatory molecules such as TNF-α, IL-1β, IL-6, MCP-1 and complement pathways [16–18]. Genetic and pharmacological inhibition of complement pathways can reduce activated microglia and attenuate white matter pathology and cognitive deficits [16] induced by cerebral hypoperfusion. Similarly, other broad-spectrum anti-inflammatory approaches can reduce white matter pathology and protect white matter function and cognitive deficits in models of cerebral hypoperfusion [14, 19]. Inflammation causes oxidative stress which is also proposed to contribute mechanistically to white matter damage and can in turn exacerbate inflammation [20, 21]. Markers of oxidative damage to proteins, lipid and nucleic acids and increased levels of free radicals have been reported in models of chronic hypoperfusion [22–25]. Oxidative stress induced by chronic hypoperfusion may be mediated by decreased levels of antioxidant enzymes such as SOD, catalase and glutathione, through mitochondrial dysfunction or indirectly via free radical production from increased levels of inflammatory cells [26–28].

The transcription factor nuclear factor erythroid 2-related factor (Nrf2) is a master regulator of endogenous cytoprotective antioxidant and anti-inflammatory signalling pathways [29]. Our work and that of others have demonstrated that cerebral hypoperfusion can activate Nrf2 signalling [30–32]. Consistent with a mechanistic role for inflammation and oxidative stress in causing white matter damage after chronic hypoperfusion, boosting Nrf2 signalling has protective effects in models of chronic cerebral hypoperfusion. Pharmacological approaches to activate Nrf2 signalling, including dimethyl fumarate and sulforaphane, protect white matter function and cognitive deficits induced by cerebral hypoperfusion [15, 33]. Furthermore, other protective approaches in models of cerebral hypoperfusion such as environment enrichment and olfactory ensheathing cell transplantation are reported to act by activating Nrf2 signalling [34, 35]. We reported a role for astrocytic Nrf2 in protecting white matter and behavioural deficits following chronic cerebral hypoperfusion in mice [36]; these protective effects were paralleled by attenuated astrogliosis and pro-inflammatory gene expression.

Conversely, lack of Nrf2 has been shown to exacerbate pathology and functional outcome in other models of neurodegenerative diseases that, in common with chronic hypoperfusion, feature oxidative stress and inflammation as disease mechanisms. These include models of Parkinson’s disease [37], familial amyotrophic lateral sclerosis [38] and AD [39–42]. In models of white matter disease (sciatic nerve injury and EAE), lack of Nrf2 impairs re-myelination and functional recovery [43, 44] indicating that Nrf2 is involved in maintaining white matter during pathological conditions and promoting white matter repair. Furthermore, Nrf2 has been shown to play a role in maintaining white matter structural integrity under normal physiological conditions, as Nrf2 knock out mice display age-related white matter disruption [45]. Collectively, these studies suggest that Nrf2 plays a role regulating white matter in health and disease. However, it is presently unclear if Nrf2 knockout mice are more vulnerable to the pathology and cognitive deficits following chronic cerebral hypoperfusion. Therefore, in the present study, we hypothesised that in mice deficient in Nrf2, white matter pathology and behavioural deficits would be exacerbated following chronic hypoperfusion.

## Methods

### Animals

All experiments were conducted in accordance with the Animal (Scientific Procedures) Act 1986 and local ethical approval at the University of Edinburgh and were performed under personal and project licences granted by the UK Home Office according to ARRIVE guidelines. Nrf2 knockout mice were originally developed by Chan et al. [46]. The Nrf2 gene was knocked out in embryonic stem cells (129X1SvJ) by homologous recombination. Adult male and female Nrf2 knockout mice (homozygous and heterozygous for Nrf2 knockout) were obtained from the Jackson Laboratory (strain B6.129X1-Nfe2l2^tm1Ywk^/J stock number 017009; backcrossed onto C57Bl/6 mice for at least 10 generations). Imported male and female homozygous Nrf2^−/−^ mice were bred with each other whereas Nrf2^+/−^ were bred with wild-type C57Bl/6Jax mice. First generation mice from both Nrf2^−/+^ and Nrf2^−/−^ breeding pairs were used for experiments. In accordance with information available from Jackson (https://www.jax.org/strain/017009), Nrf2^−/−^ were poor breeders, and limited numbers of male Nrf2^−/−^ were produced; therefore, a mix of Nrf2^+/−^ and Nrf2^−/−^ mice was used for experiments. Mice were initially group housed on a 12-h light/dark cycle with ad libitum access to food and water and assigned experimental groups by genotype then randomly assigned surgery; wild-type sham (*n* = 7), wild-type hypoperfused (*n* = 10), Nrf2^+/−^ sham (*n* = 7), Nrf2^+/−^ hypoperfused (*n* = 7), Nrf2^−/−^ sham (*n* = 3), Nrf2^−/−^ hypoperfused (*n* = 3). All mice were male, mean age 6 months (25–44 g). Three animals tolerated surgery poorly and had to be culled (2 wild-type hypoperfused, 1 Nrf2^+/−^ hypoperfused). Experimenters were blind to genotype and surgery status of the mice throughout data collection and analysis.

### Bilateral carotid artery stenosis

Bilateral carotid artery stenosis (BCAS) surgery was carried out as previously described [11]. All surgical procedures were conducted using aseptic techniques while under isoflurane anaesthesia (5% induction, 1.5% maintenance). In brief, a small cervical midline incision was made, and the common carotid arteries were exposed. A 0.18-mm internal diameter microcoil (Sawane Spring Co, Japan) was placed around one common carotid artery; the animal was allowed to recover and was 30 min later re-anesthetised for the placement of the second microcoil (0.18 mm). Sham surgery was performed in the same manner excluding the placement of the microcoils.

### Eight-arm radial arm maze to assess behavioural alterations

Behavioural testing commenced 4 weeks post-BCAS. Animals were singly housed and food restricted (maintained at 85% of initial body weight) 1 week prior to and throughout the radial arm maze test to promote motivation (12-h light/dark cycle, ad libitum access to water). Following the last trial, animals were again provided food ad libitum.

The radial arm maze comprises a central platform (20 cm in diameter) surrounded by 8 arms (47-cm long by 7-cm wide with 20-cm Plexiglas walls). Each arm has a 2-cm deep plastic well for placement of a sugar pellet, and all arms can be isolated from the central platform by Plexiglas doors (remotely controlled using the ANY-Maze software, Stoelting, UK). Large visual cues were placed on each of the four walls surrounding the maze, and a camera mounted on the ceiling was used for data acquisition (ANY-Maze software, Stoelting, UK).

Pre-training consisted of one 5-min trial of free exploration with sugar pellets scattered at random, and one where each animal was allowed to walk down each arm from the central platform to retrieve sugar pellets from the plastic cups. The training was carried out for 16 consecutive days (1 trial/day). Each arm was baited with a sugar pellet, and the animal was placed in the central platform at the start of the trial. The animal was confined to the central platform for 5 s between each arm choice, and the trial finished when the animal had retrieved all 8 pellets or when 25 min had elapsed. The number of revisiting errors (visits into unbaited arms) during each trial were recorded and analysed as a measure of behaviour.

Nrf2^+/−^ and Nrf2^−/−^ were grouped together for radial arm maze analysis. Animals that explored less than 75% of the maze during 2 of the first 4 trials were excluded from analysis due to lack of motivation resulting in skewed learning profile. Two animals were excluded according to this criterion (one wild type BCAS and one Nrf2 ^−/−^ sham). Therefore, final group sizes for behavioural analysis were wild-type sham (*n* = 7), wild-type BCAS (*n* = 7), Nrf2-deficient sham (Nrf2^+/−^ *n* = 7, Nrf2^−/−^ *n* = 2), Nrf2-deficient BCAS (Nrf2^+/−^ *n* = 6, Nrf2^−/−^ *n* = 3).

### Immunohistochemistry

Six weeks after the onset of BCAS, mice were culled under deep anaesthesia by transcardiac perfusion, and hemibrains were snap frozen in liquid nitrogen for qPCR or post-fixed in 4% paraformaldehyde for 24 h, placed in 30% sucrose for 72 h, then frozen in isopentane at − 42 °C for 2 min. Coronal sections (30 μm) were cut using a cryostat and stored in a cryoprotective medium at − 20 °C. Sections were washed in PBS (3 × 15 mins), TB (2 × 15 mins) then mounted onto Superfrost slides (VWR International) and air dried then dehydrated through a series of alcohols (70%, 90%, 100%) then placed in xylene for 10 min. Sections were then placed in 100% ethanol, and endogenous peroxidases were quenched in 3% hydrogen peroxide in methanol. Antigen retrieval was undertaken for Iba1 and GFAP immunohistochemistry; sections were incubated in 10 mM citric acid buffer (pH 6.0) at 95 °C for 10 min. Sections were blocked with 10% normal serum and 0.5% bovine albumin serum before overnight incubation with primary antibody at 4 °C. Following block, immunoglobulin G (IgG) immunostained sections were incubated overnight with horse anti-mouse IgG antibody (Vector Labs BA-2000; 1:2500). Primary antibodies and concentrations were as follows: anti-myelin-associated glycoprotein (MAG; Abcam ab89780 1:15000); anti-glial fibrillary acidic protein (GFAP; Life technologies 13-0300 1:1000); anti-ionised calcium-binding adaptor molecule-1 (Iba-1; Menarini MP290-CR05 1:1000). The appropriate biotinylated secondary antibody (Vector Labs) was incubated for 1 h at room temperature and then further amplified by incubating with Vector ABC solution before visualisation of peroxidase activity using 3,3′-diaminobenzidine tetrahydrochloride (DAB, Vector Labs, UK). IgG immunostained sections were counterstained with haematoxylin to aid region of interest analysis. Sections were then dehydrated through a series of alcohols, placed in xylene then coverslips mounted. Images were acquired using a BX51 microscope (Olympus, UK) or a Zeiss Axio Scope.A1 (Zeiss, UK). Images were analysed for percentage area of MAG, Iba1, GFAP and IgG as described in the corpus callosum, internal capsule and optic tract which were manually delineated by the experimenter. Briefly, the ImageJ software (v1.46, NIH, Bethesda, MD, USA) was used to apply a global manual threshold followed by quantification of the positive signal detected above the selected threshold. IgG immunostained sections underwent colour deconvolution in Image J (H&E DAB) to remove blue counterstained nuclei, and the Renyi Entropy threshold was applied. Occasionally, animals were excluded from analysis as the anatomical area was missing from the slide (1 wild-type BCAS and 1 Nrf2^+/−^ animal omitted from Iba1 analyses of internal capsule and optic tract; 1 wild-type BCAS animal omitted from GFAP internal capsule analysis).

### RNA extraction, reverse transcription-PCR, and quantitative (q)-PCR

RNA was isolated from optic tract-enriched samples dissected from snap-frozen hemibrains. RNA was extracted using the QIAGEN RNeasy Lipid Tissue Mini Kit according to manufacturer’s instruction. Briefly, < 100 mg fresh frozen tissue was homogenised in 1 ml QIAzol® lysis reagent using the Qiagen automated tissue lyser system and metal beads. The homogenate was transferred to fresh RNase/DNase free tubes and incubated for 2 min at room temperature with 200 μl chloroform. The upper aqueous phase was collected following 15 min centrifugation at 4 °C (12,000×*g*), and RNA was subsequently purified in mini spin columns and washed with a series of buffers before it was eluted in RNase free water. RNase-free DNase I (Thermo Scientific/QIAGEN) was used to remove genomic DNA according to the manufacturer’s instruction. cDNA was synthesised from 1 to 3 μg RNA using the Roche Transcriptor First Strand cDNA Synthesis Kit, according to manufacturer’s instruction. Briefly, RNA was added to reverse transcriptase reaction mix and cycled through 10 min 25 °C, 30 min 55 °C and 5 min 85 °C. No RT control was run alongside, and cDNA was diluted to the equivalent of 3 ng initial RNA per 15 μl qPCR reaction. The CFX96 Real-Time PCR Machine (Bio Rad) was used with the DyNAmo ColorFlash SYBR Green qPCR kit according to manufacturer’s instructions (Thermo Scientific). cDNA template was mixed with SYBR green master mix, water and forward and reverse primer (200 nM each final concentration). Samples were run in duplicates alongside no template and no RT negative controls. Primers were validated to confirm efficiency prior to use, and sequences used are as follows: *Gapdh*-F 5′-GGGTGTAACCACGAGAAAT-3′ *Gapdh*-R 5′-CCTTCCACAATGCCAAAGTT-3′ *Nrf2*-F 5′-CAGCTCAAGGGCACAGTGC-3′ *Nrf2*-R 5′-GTGGCCCAAGTCTTGCTCC-3′ *Slc7a11*-F 5′-ATACTCCAGAACACGGGCAG-3′ *Slc7a11*-R 5′-AGTTCCACCCAGACTCGAAC-3′ *Gclm-*F 5′-GCACAGCGAGGAGCTTC-3′ *Gclm-*R 5′-GAGCATGCCATGTCAACTG-3′ *Ccl3*-F 5′-GCCAGGTGTCATTTTCCTGACT-3′ *Ccl3*-R 5′-TCAGGCATTCAGTTCCAGGTC-3′ *C1q*-F a 5′-CAAGGACTGAAGGGCGTGAA-3′ *C1q*-R 5′-CAAGCGTCATTGGGTTCTGC-3′ *C4*-F 5′-ACAACAAGGGAGACCCCCAG-3′ *C4*-R 5′-GCTCAGAGAGCCAGAGTCCTA-3′.

The qPCR cycling programme was 10 min at 95 °C; 40 cycles of 30 s at 95 °C, 30 s at 65 °C (/30 s at 62.5 °C for *C1q* and *Ccl2* experiment/40 s at 60 °C for *Gclm* experiment, 62.5 °C) with detection of fluorescence, 30 s at 72 °C; 1 cycle (for dissociation curve) of 1 min at 95 °C and 30 s at 55 °C with a ramp up to 30 s at 95 °C with continuous detection of fluorescence. The *C4* experiment was run at 7 min at 95 °C initially and then 40 cycles of 10 s at 95 °C and 30 s at 65 °C (/30 s at 60 °C for *Ccl3* experiment) with detection of fluorescence followed by the dissociation curve. Data was normalised to *Gapdh* expression as reference and expressed as fold change of wild-type sham expression.

Due to the very small volumes of optic tract-enriched RNA samples, more variation was present in these qPCR experiments. Criteria were therefore defined to exclude samples from qPCR analysis if housekeeper gene was detected > 3Ct away from the mean or if gene of interest expression was 1.5 interquartile ranges less than the first quartile or 1.5 interquartile ranges more than the third quartile for each group mean. The *Ccl3* experiment therefore had one sample excluded (wild-type sham), and the Slc7a11 experiment had one sample excluded (Nrf2^+/−^ BCAS). All other qPCR experiments included all samples.

### Statistical analysis

Statistical analysis was performed using SPSS (v22, IBM Corp.) or Graphpad Prism (v5, GraphPad Software Inc, La Jolla, USA). Data are presented as mean ± SEM. Repeated measures ANOVA was used to analyse radial arm maze data. Statistical analysis was performed on the first and second half of the radial arm maze separately because the first half is primarily a learning phase. Two-way ANOVA was used to investigate the effect of BCAS surgery and genotype on immunohistochemistry and gene expression data. Bonferroni adjustment was used for post hoc analysis. Associations between microglial density and white matter pathology were analysed with Pearson’s correlation analysis. Significance was determined at *p* < 0.05.

## Results

### White matter pathology is more extensive in Nrf2-deficient mice compared to wild-type mice post-BCAS

**Fig. 1:**
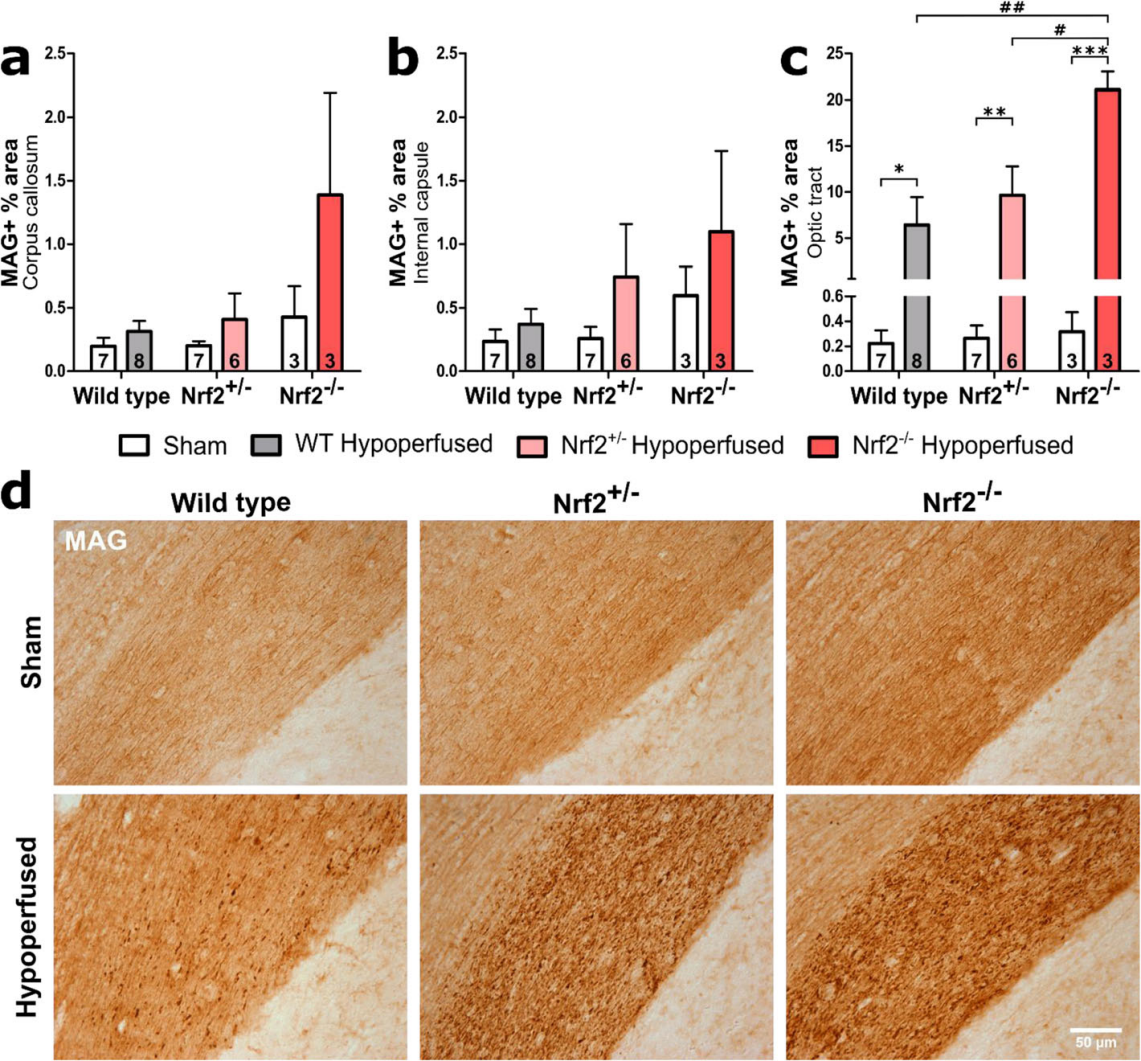
White matter disruption is more extensive in Nrf2-deficient mice following BCAS surgery. **a** White matter disruption was detected post-BCAS in the corpus callosum, with a significant effect of genotype (no post hoc differences). **b** There was no significant alteration in the levels of white matter disruption in the internal capsule when comparing experimental groups. **c** The optic tract displayed prominent white matter disruption post-BCAS with a further effect of genotype, with the Nrf2^−/−^ group displaying the most severe disruption. Mean ± SEM. Group size presented in each bar. *p < 0.05, **p < 0.01, ***p < 0.001 (asterisk indicates post hoc differences between sham and BCAS), #*p* < 0.05, ##*p* < 0.01 (number sign indicates post hoc differences between BCAS groups). **d** Representative images of MAG immunostaining in the optic tract. Scale bar 50 μm

We previously demonstrated alterations in the intensity of myelin-associated glycoprotein (MAG) immunostaining in response to chronic cerebral hypoperfusion that is indicative of altered axon-glial integrity [11] and associated with alterations in white matter integrity assessed with diffusion tensor imaging [10]. To determine if deficiency of Nrf2 exacerbated MAG pathology, the percentage area of MAG immunostaining was quantified in key white matter tracts: corpus callosum, internal capsule and optic tract (Fig. 1). There was significant white matter disruption in the corpus callosum following BCAS (*F*(_1,28_) = 6.04, *p* = 0.02) with an additional effect of genotype (*F*(_2,28_) = 4.47, *p* = 0.02), but no interaction or significant post hoc effects (Fig. 1a). There was no significant white matter disruption in the internal capsule as a result of BCAS surgery or genotype (*F*(_1,28_) = 2.99, *p* = 0.09, *F*(_2,28_) = 1.89, *p* = 0.17 respectively) (Fig. 1b). Notably in the optic tract, there was prominent white matter disruption post-BCAS (*F*(_1,28_) = 35.84, *p* < 0.0001) with a further effect of genotype (*F*(_2,28_) = 3.96, *p* = 0.03) and a significant interaction between the two variables (*F*(_2,28_) = 3.87, *p* = 0.03) (Fig. 1c). Post hoc analysis found significant differences between all BCAS groups and their respective sham controls (WT *p* = 0.04, Nrf2^+/−^ *p* = 0.004, Nrf2^−/−^ *p* < 0.001). Furthermore, there was significantly greater white matter disruption in the Nrf2^−/−^ BCAS group compared to both Nrf2^+/−^ (*p* = 0.02) and wild-type BCAS (*p* = 0.001) (Fig. 1c). Collectively, these data show that cerebral hypoperfusion induced by BCAS surgery causes white matter disruption in two major white matter tracts and that this disruption is exacerbated in the absence of Nrf2.

### The density of microglia/macrophages is increased following BCAS to a greater extent in Nrf2^−/−^ mice compared to wild-type and Nrf2^+/−^ mice

**Fig. 2:**
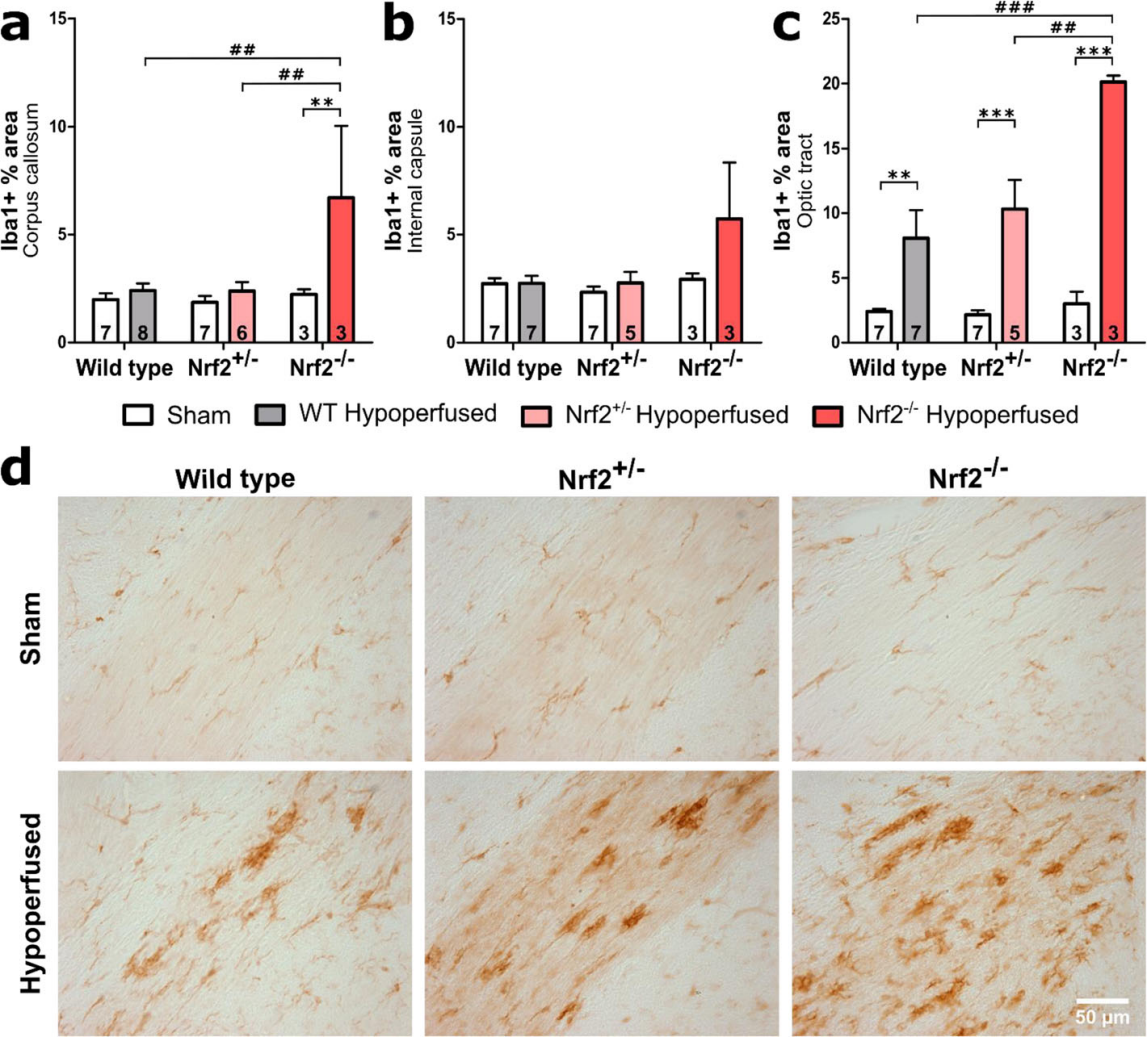
The density of microglia/macrophages is increased after BCAS to a greater extent in Nrf2^−/−^ mice. There was an effect of BCAS surgery and genotype on density of Iba1 in the corpus callosum, with the Nrf2^−/−^ BCAS group displaying the highest density. **b** The effect of BCAS and genotype narrowly missed accepted levels of statistical significance in the internal capsule. **c** The optic tract displayed an increased density of Iba1 post-BCAS, with a further effect of genotype, with the Nrf2^−/−^ BCAS group displaying the highest density. Mean ± SEM. Group size presented in each bar. **p < 0.01, ***p < 0.001 (asterisk indicates post hoc differences between sham and BCAS), #*p* < 0.05, ##*p* < 0.01, ###*p* < 0.001 (number sign indicates post hoc differences between BCAS groups). **d** Representative images of Iba1 staining in the optic tract. Scale bar 50 μm

We previously reported that deficits in white matter function and pathology following chronic cerebral hypoperfusion was associated with increased density of microglia/macrophages [14, 15] Similarly, Nrf2^−/−^ mice have been reported to have an increased number of microglia at baseline [47] and an exacerbated microglial response in disease models such as Parkinson’s disease [47] and Alzheimer’s disease [39–41]. Therefore, we next investigated whether deficiency of Nrf2 exacerbated levels of microglia/macrophages in response to BCAS using Iba1 immunohistochemistry. Microglia/macrophage density was quantified by assessing percentage area of Iba1 immunostaining, which is an index of microglial activation [48], in corpus callosum, internal capsule and optic tract (Fig 2, Fig S1). There was a significant increase in density of microglia post-BCAS in the corpus callosum (*F*(_1,28_) = 7.63, *p* = 0.01) with a further effect of genotype (*F*(_2,28_) = 4.25, *p* = 0.02) but no interaction (Fig. 2a). Post hoc analysis identified significantly greater density in the Nrf2^−/−^ BCAS group compared to sham controls (*p* = 0.004) and other BCAS groups (Nrf2^+/−^ *p* = 0.005 and wild types *p* = 0.003 respectively) (Fig. 2a). The effect of BCAS surgery and genotype narrowly missed accepted levels of statistical significance in the internal capsule (*F*(_1,26_) = 3.66, *p* = 0.07, *F*(_2,26_) = 3.15, *p* = 0.06 respectively) (Fig. 2b). However, similar to the white matter disruption, there was a profound microglial response in the optic tract post-BCAS (Fig. 2c). There was a significant effect of both BCAS surgery and genotype (*F*(_1,26_) = 61.12, *p* < 0.0001, *F*(_2,26_) = 7.21, p = 0.003 respectively) and a significant interaction (*F*(_2,26_) = 5.8, *p* = 0.008). Post hoc analysis found significantly greater density of microglia in all BCAS groups compared to their sham controls (WT *p* = 0.005, Nrf2^+/−^ *p* < 0.001, Nrf2^−/−^ *p* < 0.001) and in the Nrf2^−/−^ BCAS group compared to both Nrf2^+/−^ (*p* = 0.002) and wild-type BCAS (*p* < 0.001) (Fig. 2c). These data show that cerebral hypoperfusion induced by BCAS surgery causes an increased density of microglia in two major white matter tracts which exhibit white matter pathology and that this increase is exacerbated in the absence of Nrf2.

**Fig. 3:**
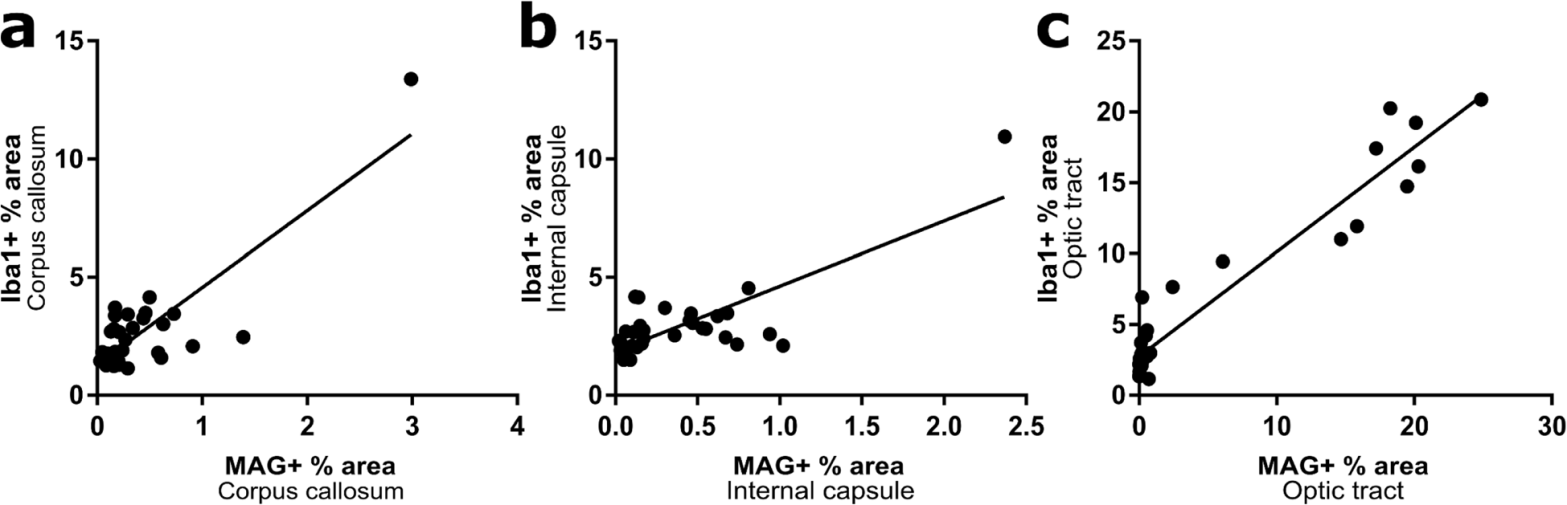
Correlation between microglial activation and white matter pathology following BCAS surgery in WT and Nrf2 deficient mice. There was a significant positive correlation between levels of Iba1 immunostaining and MAG immunostaining in the **a** corpus callosum, **b** internal capsule, **c** optic tract

We previously showed that was a correlation between impaired white matter function and microglial numbers after BCAS [14, 15], and others have suggested that aberrant activated microglia aggravate white matter injury during chronic hypoperfusion [16]. Therefore, we next investigated the association between microglial density and white matter disruption by correlating Iba1 and MAG immunostaining (Fig. 3). There was a significant positive correlation between microglial density and altered MAG disruption in the corpus callosum (*r* = 0.85, *p* < 0.0001; Fig. 3a), internal capsule (*r* = 0.78, *p* < 0.0001; Fig. 3b) and optic tract (*r* = 0.96, *p* < 0.0001; Fig. 3c). This indicates that there is an association between microglial activation and white matter pathology following BCAS surgery in WT and Nrf2-deficient mice.

### Reactive astrocytes are elevated in the optic tract following BCAS and are unaltered by deficiency of Nrf2

**Fig. 4:**
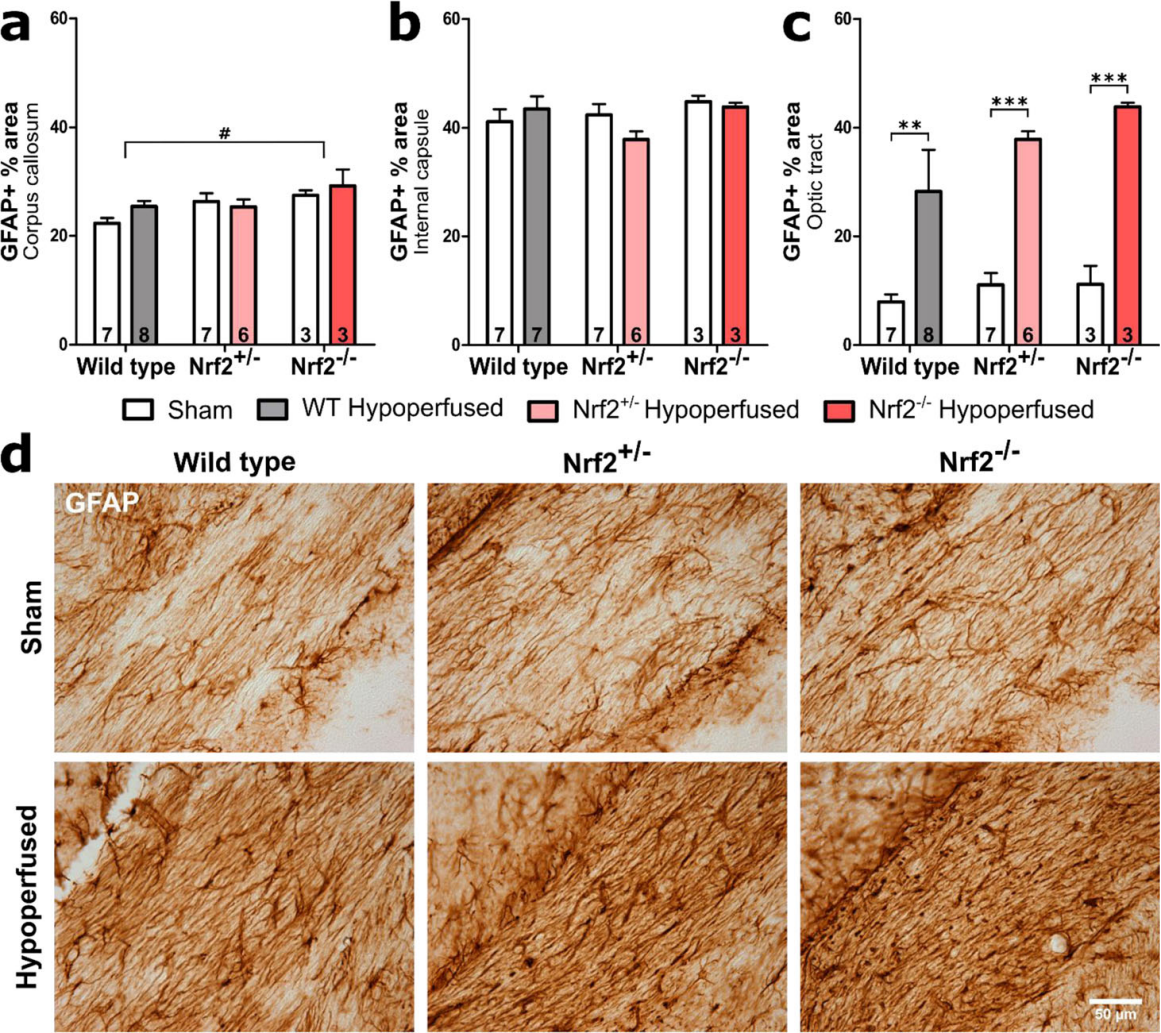
Elevated levels of reactive astrocytes after BCAS that are not exacerbated by deficiency of Nrf2. **a** There was a significant effect of genotype on GFAP immunostaining (% area) in the corpus callosum but there was no effect of BCAS surgery. **b** Percent area of GFAP was unchanged in the internal capsule. **c** There was a significant effect of BCAS surgery on percent area of GFAP immunostaining in the optic tract, but the effect of genotype narrowly missed accepted levels of statistical significance. Mean ± SEM. **p < 0.01, ***p < 0.001 (asterisk indicates post hoc differences between sham and BCAS), #*p* < 0.05 (number sign indicates post hoc differences between wild type and Nrf2^−/−^). Group sizes presented in each bar. **d** Representative images of GFAP staining in the optic tract. Scale bar 50 μm

Aged Nrf2 knockout mice display elevated levels of reactive astrocytes in white matter compared with wild-type aged mice [45]. Similarly, elevated levels of reactive astrocytes are reported with Nrf2 deficiency in models of Parkinson’s disease [47] and Alzheimer’s disease [39]. Therefore, we investigated the effects of Nrf2 deficiency on levels of reactive astrocytes following chronic cerebral hypoperfusion by undertaking GFAP immunostaining of white matter tracts (Fig. 4, Fig S2). BCAS surgery did not affect the percentage area of GFAP immunostaining in the corpus callosum or the internal capsule (*F*(_1,28_) = 1.08, *p* = 0.31; *F*(_1,27_) = 0.30, *p* = 0.59 respectively) (Fig. 4a, b). However, there was an effect of genotype in the corpus callosum (*F*(_2,28_) = 3.96, *p* = 0.03), and post hoc analysis found that GFAP expression in Nrf2^−/−^ animals was overall higher compared with wild types (*p* = 0.03). This genotype effect was not present in the internal capsule (*F*(_2,27_) = 1.52, *p* = 0.24). Consistent with the white matter disruption and the microglial response, there was a significant effect of BCAS surgery in the optic tract (*F*(_1,28_) = 43.61, *p* < 0.0001), although the effect of genotype narrowly missed accepted levels of statistical significance (*F*(_2,28_) = 3.26, *p* = 0.053) (Fig. 4c). Post hoc analysis found significant astrogliosis in all three BCAS groups compared to their sham controls (WT *p* = 0.005, Nrf2^+/−^ *p* < 0.001, Nrf2^−/−^ *p* < 0.001). These data show that Nrf2 deficiency but not hypoperfusion exacerbated levels of reactive astrocytes in the corpus callosum, whereas cerebral hypoperfusion induced by BCAS surgery causes astrogliosis in the optic tract that was not altered with deficiency of Nrf2.

### Blood-brain barrier breakdown is increased following BCAS to a greater extent in Nrf2^−/−^ mice compared to wild-type and Nrf2^+/−^ mice

**Fig. 5:**
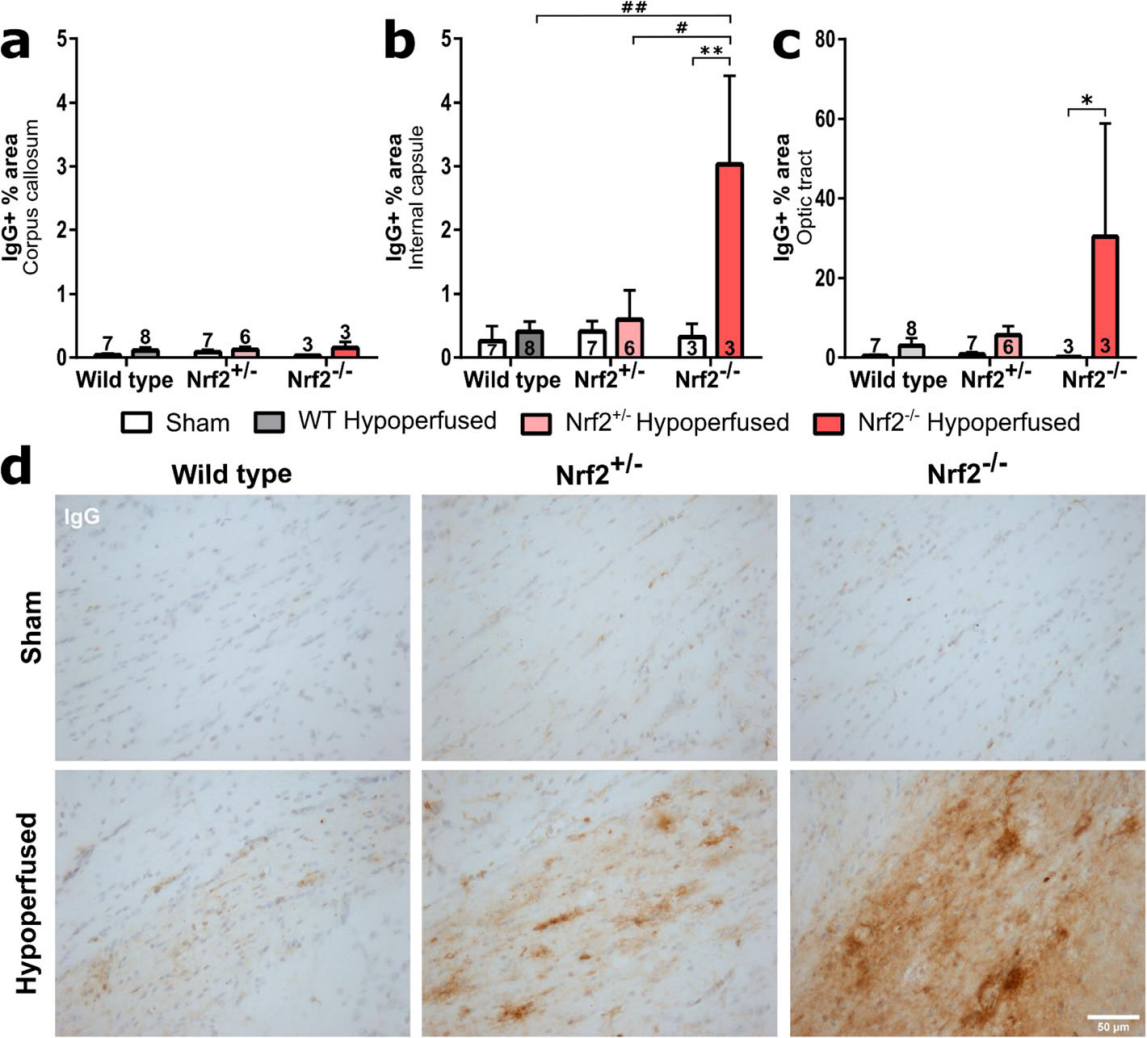
Blood-brain barrier breakdown after BCAS is exacerbated by Nrf2 deficiency. **a** There were no significant alterations in IgG levels in the corpus callosum. **b** There was a significant effect of surgery and genotype on IgG levels in the internal capsule, with Nrf2^−/−^ displaying highest density of IgG. (Double asterisk indicates post hoc significant difference between sham and BCAS Nrf2^−/−^ mice, double number sign and number sign indicate a difference between Nrf2^−/−^ BCAS and wild type or Nrf2^+/−^ BCAS groups respectively) **c** There was a significant increase in IgG levels in the optic tract with BCAS surgery (asterisk indicates post hoc significant difference between sham and BCAS Nrf2^−/−^ mice). Mean ± SEM. **p < 0.01, #*p* < 0.05, ##*p* < 0.01. **d** Representative images of IgG staining in the optic tract, brown stain depicts IgG, blue is haematoxylin counterstain. Scale bar 50 μm

We previously showed that BCAS induced blood-brain barrier breakdown (BBB), although this occurred in the chronic response to surgery (6 months) and was not evident at earlier timepoints (1 month) [13]. Because Nrf2 deficiency exacerbated white matter pathology and microglial/macrophage levels, we next investigated if this induced elevated levels of blood-brain barrier breakdown by labelling endogenous immunoglobulin G (IgG) within brain tissue, which would normally be excluded by an intact BBB [49] (Fig. 5). BCAS surgery did not induce blood-brain barrier breakdown in the corpus callosum (Fig. 5a) (*F*(_1,28_) = 0.24, *p* = 0.068), which contained negligible levels of IgG immunostaining that were not altered by Nrf2 deficiency (*F*(_2,28_) = 0.24, = 0.79). In contrast, in the internal capsule (Fig. 5b), there was a significant increase in IgG levels with BCAS surgery (*F*(_1,28_) = 8.99, *p* = 0.006), an effect of genotype (*F*(_2,28_) = 4.9, *p* = 0.015) and a significant interaction (*F*(_2,28_) = 4.8, *p* = 0.016). Post hoc analysis found a significantly greater density of IgG in the Nrf2^−/−^ BCAS group compared to its sham control (*p* < 0.05) and in the Nrf2^−/−^ BCAS group compared to both Nrf2^+/−^ (*p* < 0.05) and wild-type BCAS (*p* < 0.01) (Fig. 5b). BCAS surgery also caused a significant elevation in IgG levels in the optic tract (Fig. 5c) (*F*(_1,28_) = 6.02, *p* = 0.02), although there was no effect of genotype (*F*(_2,28_) = 2.2, *p* = 0.13). Post hoc analysis found a significant increase in IgG levels in the Nrf2^−/−^ BCAS group compared to its sham control (*p* < 0.05). Collectively, these data show that BCAS surgery causes blood-brain barrier breakdown in the internal capsule and optic tract, an effect that is exacerbated with deficiency of Nrf2.

### Nrf2 and Nrf2-related antioxidant genes are unaltered post-BCAS

**Fig. 6:**
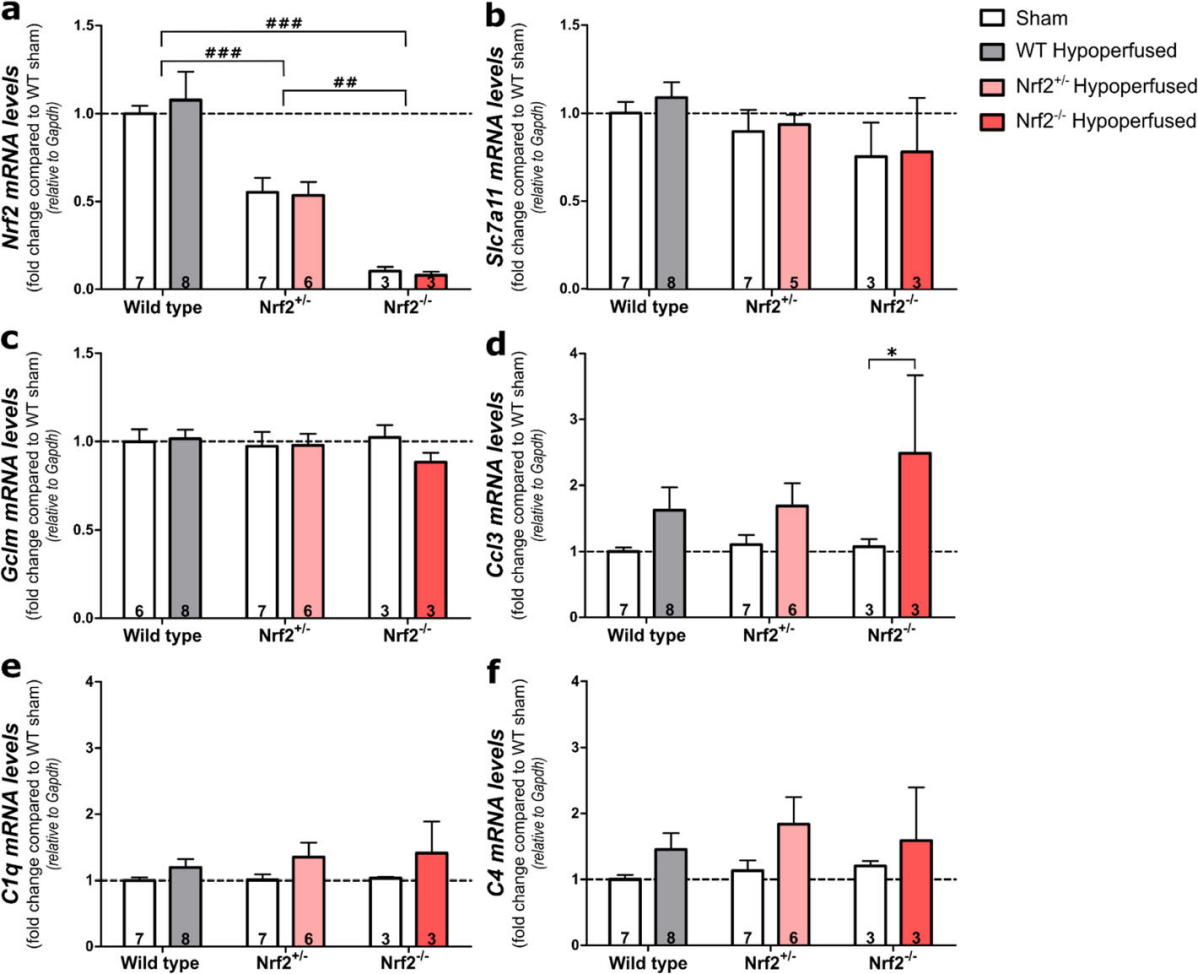
Nrf2, Nrf-2-regulated antioxidant and pro-inflammatory genes in the optic tract after BCAS surgery. **a** There was a significant effect of genotype on Nrf2 expression but no effect of BCAS surgery. Nrf2 was reduced by ~ 50% in Nrf2^+/−^ mice and almost undetectable in Nrf2^−/−^ mice. **b** There was no effect of BCAS surgery or genotype on *Slc7a11* or **c** *Gclm* expression; however, BCAS surgery increased the expression of **d** *Ccl3* and **e** *C1q*. **f** The effect of BCAS surgery just missed accepted levels of statistical significance for *C4* expression and there was no genotype effect on any gene. Gene expression was normalised to Gapdh and expressed as fold change compared to wild-type shams. Mean ± SEM. Group size presented in each bar. Dashed line indicates WT sham mean. *p < 0.05 (asterisk indicates post hoc differences between sham and BCAS groups), ##*p* < 0.01, ###*p* < 0.001 (number sign indicates differences between genotypes)

To confirm that Nrf2 levels were reduced in Nrf2-deficient mice, and to determine if BCAS surgery altered Nrf2 levels in the experimental groups, quantitative PCR analysis was used to assess gene expression levels (Fig. 6). Optic tract-enriched samples were used, as this was the area with most prominent pathology. As expected, Nrf2 levels were significantly reduced to about 50% in Nrf2^+/−^ mice (*p* < 0.001) and nearly undetectable in Nrf2^−/−^ compared to wild-type mice (*p* < 0.001) (genotype effect; *F*(_2,28_) = 29.6, *p* < 0.0001) (Fig. 6a). However, BCAS surgery did not alter the expression of Nrf2 in either WT or Nrf2-deficient mice (*F*(_1,28_) = 0.01, *p* = 0.91). To further investigate the effect of BCAS surgery on Nrf2-mediated signalling, two genes regulated by Nrf2-signalling that are involved in glutathione synthesis were investigated, *Slc7a11* (xCT; encoding the glutamate/cystine antiporter) and *Gclm*, (glutamate-cysteine ligase enzyme subunit) that were previously reported to play a role in Nrf2-mediated neuroprotection against ischaemia and hypoxia [30, 31, 50]. However, there was no effect of BCAS surgery or genotype on *Slc7a11* levels (*F*(_1,27_) = 0.23, *p* = 0.63, *F*(_1,27_) = 2.25, *p* = 0.13 respectively) or *Gclm* expression (*F*(_1,27_) = 0.39, *p* = 0.54, *F*(_1,27_) = 0.24 *p* = 0.79 respectively) (Fig. 6 b, c). Collectively, these results suggest that Nrf2 was reduced as expected in Nrf2-deficient mice, but Nrf2 and Nrf2-regulated genes (*Slc7a11* and *Gclm*) were not altered in wild-type or Nrf2-deficient mice in response to BCAS.

Therefore, to further investigate alternative mechanisms that may be responsible for the elevated white matter pathology in Nrf2-deficient mice in response to BCAS, we investigated levels of 3 pro-inflammatory genes, complement component *C4* and *C1q* and chemokine ligand 3 (*Ccl3*) that we previously found to be elevated in response to hypoperfusion [9, 15]. The expression of *Ccl3* and *C1q* was increased post-BCAS (*F*(_1,28_) = 7.96, *p* = 0.009, *F*(_1,28_) = 4.61, *p* = 0.04 respectively), whereas the effect of BCAS surgery on *C4* expression narrowly missed accepted levels of statistical significance (*F*(_1,28_) = 3.9, *p* = 0.058) (Fig. 6d–f). However, there was no significant effect of genotype for these 3 genes (*Ccl3*-*F*(_2,28_) = 0.68, *p* = 0.52; *C1q-F*(_2,28_) = 0.28, *p* = 0.76; *C4*-*F*(_2,28_) = 0.48, *p* = 0.63), although post hoc analysis identified significantly increased expression of *Ccl3* in the Nrf2^−/−^ BCAS group compared to sham controls (*p* = 0.048). These data show that BCAS surgery caused modest elevations in pro-inflammatory gene expression that were not altered with Nrf2 deficiency with the exception of the chemokine ligand C3 that was further elevated in Nrf2^−/−^ mice following BCAS.

### Behaviour assessed with radial arm maze is impaired post-BCAS but is not exacerbated by deficiency of Nrf2

**Fig. 7:**
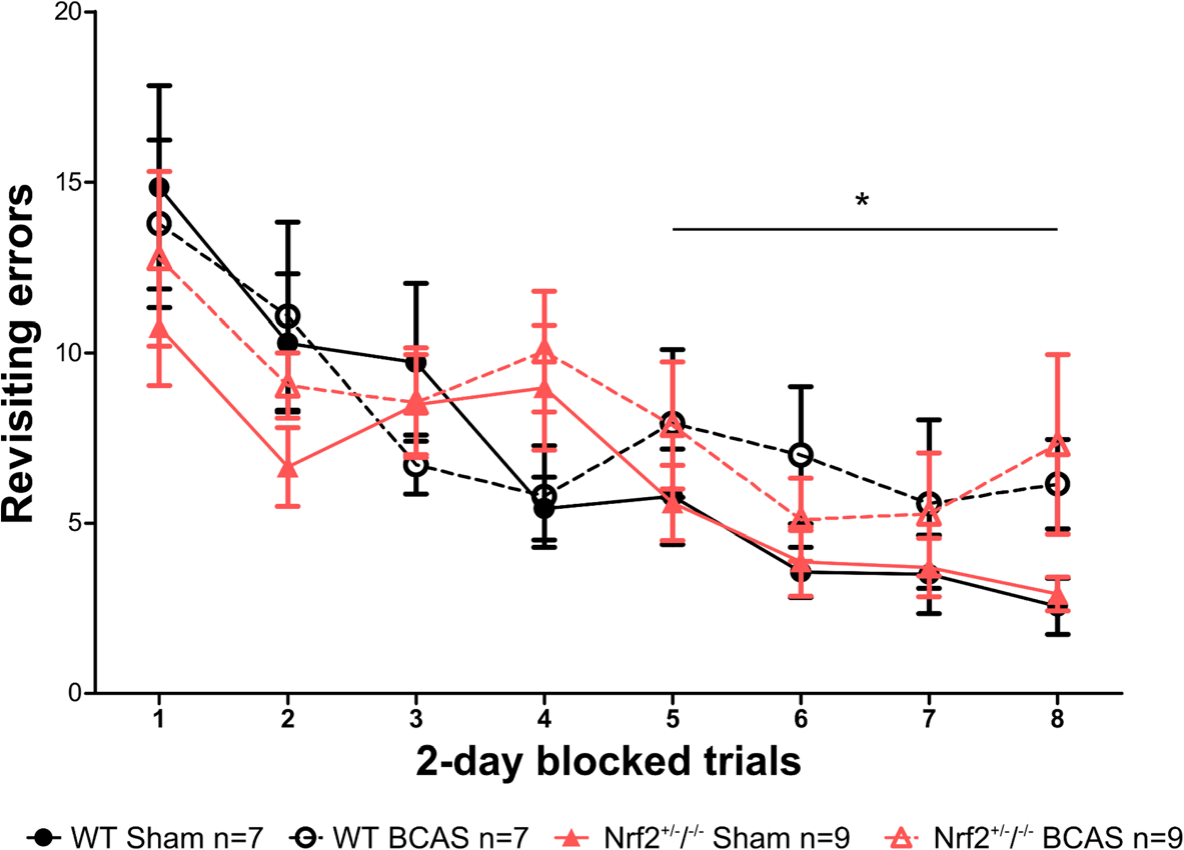
Behaviour assessed with radial arm maze is impaired after BCAS but is not exacerbated by deficiency in Nrf2. There was an effect of trial during both first (blocks 1–4) and second half (blocks 5–8) of the test indicating learning over time. During the second half of the test, revisiting errors were significantly higher post-BCAS demonstrating impaired spatial working memory. However, there was no effect of genotype on revisiting errors during either the first or second part of the test. Post hoc analyses identified no further significant differences. Mean ± SEM. *p < 0.05 (asterisk indicates main effect of BCAS surgery during second half of test). Group sizes are indicated in the graph

We previously showed that chronic cerebral hypoperfusion caused an impairment in behavioural performance assessed with an 8-arm radial arm maze, due to the disruption of frontocortical circuity [11], and furthermore, boosting Nrf2 signalling specifically in astrocytes caused less pronounced behavioural impairments [36]. Since white matter pathology is exacerbated in Nrf2-deficient mice, the next aim was to determine if this was associated with more severe behavioural impairment. There was a significant effect of trial during both the first and second half of the test (*F*(_1,84_) = 7.03, *p* < 0.0001, *F*(_3,84_) = 3.62, *p* = 0.02 respectively) indicating that mice were able to learn the task successfully (Fig. 7). The effect of BCAS surgery was not significant during the first half of the test (*F*(_1,28_) = 0.10, *p* = 0.76) but was significant during the second half (*F*(_1,28_) = 4.21, *p* = 0.049) (Fig. 7). This indicates that while both sham and BCAS groups were learning the task initially, the BCAS groups’ learning plateaus, and they commit significantly more revisiting errors than sham groups during the second half of the test, indicating that BCAS caused significantly more revisiting errors. However, there was no effect of genotype on spatial working memory (first half, *F*(_1,28_) = 0.07, *p* = 0.79; second half, *F*(_1,28_) = 0.001, *p* = 0.98) (Fig. 7), which contests the hypothesis that reductions in Nrf2 exacerbates the behavioural impairment caused by BCAS-induced cerebral hypoperfusion.

## Discussion

In the current study, we demonstrated that deficiency of Nrf2 resulted in exacerbated white matter pathology in response to chronic hypoperfusion, an effect that was most marked with complete absence of Nrf2. Furthermore, this was associated with elevated levels of microgliosis and paralleled by elevated levels of blood-brain barrier breakdown. Although Nrf2-related antioxidant gene expression was not altered by chronic cerebral hypoperfusion, there was evidence for elevated pro-inflammatory-related gene expression following hypoperfusion that was not affected by Nrf2 deficiency. However, despite these molecular and pathological alterations post-BCAS in Nrf2 deficient mice, there was no worsening of the behavioural impairment of mice in the radial arm maze.

We found elevated levels of white matter disruption in the present study in the corpus callosum and optic tract 6 weeks after chronic hypoperfusion, evident by increased density of MAG-positive debris. MAG is expressed at the axon-glial interface and is sensitive to hypoxia-induced injury [51]. Human post-mortem studies identify loss of MAG, in particular its ratio to the more stable proteolipid protein (PLP), as a measure of white matter ischaemia [52]. Our previous work showed that chronic cerebral hypoperfusion in mice induces alterations to MAG as early as 3 days post-surgery, and at least initially in the absence of damage using other markers of myelin such as myelin basic protein and FluoroMyelin [9, 12], consistent with hypoxia-induced white matter injury in humans [51]. In contrast to our previous work and that of others [9, 11, 53], the optic tract was most severely damaged after BCAS whereas the corpus callosum and internal capsule were minimally damaged. However, this is consistent with the pattern of damage we recently reported in mice that overexpress Nrf2 specifically in astrocytes [36]. The background strain of the mice may account for these differences, as we report diffuse damage to multiple white matter tracts using mice on a pure C57Bl6/J background, whereas the severe optic tract damage was reported using mice originating from mixed backgrounds (129X1/SvJ or FVB/C57Bl6/J F1 mice). These strains may have a different circle of Willis patency, and therefore different spatial distribution and severity of hypoperfusion [54]. In addition, we cannot discount that the heightened vulnerability of the optic tract may account for the impaired behaviour in the radial arm maze in the hypoperfused mice.

The MAG debris induced by cerebral hypoperfusion, indicative of altered axon-glial integrity, was exacerbated in the absence of Nrf2 in the corpus callosum and optic tract. This is consistent with earlier studies investigating the role of Nrf2 in the maintenance of white matter integrity in health and disease [44, 55]. Hubbs et al. [45] found that the absence of Nrf2 in aged mice (> 10 months) is associated with vacuolar leukoencephalopathy causing white matter degeneration and myelin-specific oxidative injury. Furthermore, patients with de novo missense mutations in *NFE2L2* that lead to accumulation of Nrf2 and increased expression of Nrf2-related genes present with leukoencephalopathy and diminished myelin content [56]. Therefore, it would appear that not only lack of Nrf2 but also chronic activation may cause pathology in white matter, highlighting the importance of the elaborate mechanisms that control Nrf2 activity.

White matter disruption after BCAS has previously been shown to be paralleled by cellular inflammation primarily characterised by a microglial response [9, 53, 57, 58], and here, we report correlations between white matter damage and microglial activation. White matter inflammation is proposed to contribute to age and disease-related white matter pathology through excessive pro-inflammatory signalling, phagocytosis and oxidative stress [59]. The data presented in the current study are consistent with previous work displaying increased density of microglia/macrophages in the corpus callosum, correlating with the severity of white matter functional deficit [15]. The results also demonstrate that hypoperfusion-induced increases in microglial/macrophage density are further increased in the absence of Nrf2, suggesting that signalling through Nrf2 normally acts to limit upregulation of microglia following cerebral hypoperfusion.

Microglia in white matter are important for myelin repair both through phagocytosis of myelin degradation products which are neurotoxic and prevent recovery if not cleared [52] and through release of growth factors [60]. A study of peripheral nerve crush injury found that Nrf2^−/−^ mice had a reduced ability to clear myelin debris [43]. Similarly, Rojo et al. [47] identified reduced levels of phagocytosis in Nrf2^−/−^ mice, suggesting a mechanism by which white matter disruption may be exacerbated in Nrf2^−/−^ mice in the current study. Nrf2 has been suggested to act as a regulator of microglial dynamics [47, 61].

In the present study, we showed differential effects of hypoperfusion and Nrf2 deficiency on reactive astrocyte density in white matter, depending on the anatomical area analysed. Interestingly, there was an increase in the density of GFAP-positive astrocytes in both sham and hypoperfused Nrf2^−/−^ mice in the corpus callosum. This finding is in agreement with Hubbs et al. [45] who found that aged Nrf2^−/−^ mice display widespread astrogliosis in white matter including corpus callosum.

Although levels of reactive astrocytes in the optic tract were elevated in response to chronic hypoperfusion, their levels were not altered by Nrf2 deficiency. In contrast, chronic hypoperfusion did not alter levels of reactive astrocytes in the corpus callosum or internal capsule, despite elevated levels of white matter damage and microglia/macrophage in the corpus callosum that were further increased in Nrf2 knockout mice. Similarly, in a cuprizone model of demyelination, loss of myelin was exacerbated in Nrf2-deficient mice, and although this was associated with increased microglia/macrophage levels, there was a 50% reduction in levels of reactive astrocytes when compared with wild-type cuprizone-treated mice. Importantly, alterations to levels of reactive astrocytes, as assessed with GFAP immunostaining, does not inform on the antioxidant and pro/anti-inflammatory capacity of those astrocytes, which may differ between genotypes. Transcriptomic analysis of isolated astrocytes display a different profile based on the activating insult, suggested to indicate the functional phenotype of that population of reactive astrocytes [62]. Additionally, astrocytes have also been shown to display both regional and age-dependent heterogeneity [63, 64]. Therefore, RNA sequencing of isolated astrocytes from different white matter regions would provide more comprehensive information about phenotype alterations to astrocytes with chronic hypoperfusion and Nrf2 deficiency.

As expected, the present study showed that there was a 50% reduction of *Nrf2* in Nrf2^+/−^ mice, and *Nrf2* expression was nearly undetectable in Nrf2^−/−^ mice compared to wild-type controls. However, cerebral hypoperfusion did not alter the expression of *Nrf2* in Nrf2^+/−^ or wild type mice. The glutathione system has been shown to be a major pathway mediating neuroprotection against oxidative stress by Nrf2 [50]. However, we did not report any alterations in *Slc7a11* and *Gclm*, genes required for the synthesis of glutathione, with either cerebral hypoperfusion or Nrf2 deficiency. It is possible that *Nrf2* and Nrf2-related gene expression may have increased acutely following the reduction of CBF. This is observed following middle cerebral artery occlusion where Nrf2 peaks between 8 [65] and 24 h [66] but is reduced again at 72 h. A similar phenomenon is observed following traumatic brain injury [67] and indicates that regulatory mechanisms of the Nrf2 system may prevent it from being chronically activated, particularly given the white matter lesions and neurological consequences of chronic Nrf2 elevation reported in patients with mutations in the gene encoding Nrf2 [56].

Because we did not report alterations in Nrf2 or Nrf2-related gene expression following cerebral hypoperfusion in the present study, an alternative explanation is that pro-inflammatory gene expression may be altered, consistent with the exacerbated levels of microgliosis that we report with both hypoperfusion and Nrf2 deficiency. Pro-inflammatory gene signalling is exacerbated in Nrf2^−/−^ mice in other disease models [42, 44, 47]. Here, we found elevated levels of chemokine *Ccl3* and complement component *C1q* following cerebral hypoperfusion, consistent with our previous work [12, 15, 36]. Post hoc analysis showed that *Ccl3* levels were significantly increased in Nrf2^−/−^ mice in comparison with shams. CCL3 regulates the recruitment of microglia as well as peripheral monocytes and macrophages [68]. We also reported that an Nrf2-activating drug, dimethyl fumarate, dampened levels of CCL3 (MIP-1α), an effect paralleled by decreased levels of microglia/macrophage and improved white matter function [15]. Hypoperfusion induced a significant increase in C1q, C4 expression just missed accepted levels of significance, although there was no genotype effect. Activation of the complement pathway has been suggested to potentiate chronic inflammation and neurodegeneration [69]. Interestingly, the complement cascade has also been shown to be activated by myelin-associated products [70], suggesting that white matter disruption in itself may potentiate inflammatory changes observed following cerebral hypoperfusion.

Cerebral hypoperfusion has previously been shown to cause an impairment in spatial working memory [11, 53] attributed to disruption of frontal-subcortical circuitry. Consistent with previous reports, the current study demonstrated that cerebral hypoperfusion impairs behaviour assessed with the radial arm maze both in wild-type and Nrf2-deficient mice. However, contrary to the study hypothesis, Nrf2 deficiency did not exacerbate revisiting errors despite the exacerbation of white matter damage. This contrasts with the protective effects that we demonstrated using the radial arm maze in mice with astrocyte-specific overexpression of Nrf2 that underwent cerebral hypoperfusion [36]. One limitation of the current study is the limited number of Nrf2^−/−^ mice, due to breeding problems. Therefore, it is possible that the study is underpowered to detect exacerbated white matter pathology in more subtly damaged white matter tracts that may have impacted on the behavioural performance. There is a very limited number of studies investigating cognition in models of CNS disease with Nrf2-deficient mice, with only one study [40] reporting an exacerbated spatial learning and memory deficits and working and associative memory deficits in an Nrf2-deficient APP/PS1 mutant model of Alzheimer’s disease. However, different disease mechanisms, anatomical differences in pathology and the older age of the mice may account for the increased vulnerability of these mice to cognitive deficits.

It is also possible that in the present study the exacerbation of white matter damage was of an insufficient level to worsen the behavioural alterations detected using the radial arm maze at the 6-week time point. Longer duration of hypoperfusion would be expected to intensify the pathological differences between wild-type and Nrf2-deficient animals, and a later time point in aged animals may have identified functional effects of Nrf2 deficiency. Indeed, with ageing, the cerebral blood vessels of Nrf2-deficient mice have elevated levels of senescence markers, ageing-induced vascular inflammation and blood-brain barrier leakage [71, 72] and white matter leukoencephalopathy [45].

## Conclusions

To conclude, we have demonstrated that chronic cerebral hypoperfusion induces white matter pathology, elevated levels of microglia/macrophages and blood-brain barrier breakdown that were exacerbated with deficiency of Nrf2, thus confirming a role for Nrf2 in protecting white matter from pathology and in microglial dynamics. Reactive astrocytes and pro-inflammatory gene expression were increased with cerebral hypoperfusion but were not altered by Nrf2 deficiency. Despite elevated levels of white matter pathology, there was no exacerbation of the impaired behavioural performance in the radial arm maze induced by cerebral hypoperfusion in Nrf2-deficient mice.

## Supplementary Information

**Additional file 1.** Supplemental figures

## Data Availability

The datasets generated and/or analysed during the current study are available from the corresponding author on reasonable request.

## Abbreviations

AD: Alzheimer’s disease
BBB: Blood-brain barrier
BCAS: Bilateral common carotid artery stenosis
C1q: Complement component 1q
C4: Complement component 4
Ccl3: Chemokine ligand 3
EAE: Experimental autoimmune encephalomyelitis
GFAP: Glial fibrillary acidic protein
Iba1: Ionised calcium-binding adaptor molecule-1
IgG: Immunoglobulin G
IL-1β: Interleukin 1-beta
IL-6: Interleukin 6
MAG: Myelin-associated glycoprotein
MCP-1: Monocyte chemoattractant protein-1 (also called CCL2 = chemokine ligand 2).
Nrf2: Nuclear factor (erythroid-derived 2)-like 2
PBS: Phosphate-buffered saline
PFA: Paraformaldehyde
ROI: Region of interest
SOD: Superoxide dismutase
TNF-α: Tumour necrosis factor alpha
VCI: Vascular cognitive impairment
