# Supplementary figures and images for: Deficiency of Nrf2 exacerbates white matter damage and microglia/macrophage levels in a mouse model of vascular cognitive impairment
Source: J Neuroinflammation. 2020 Dec 1;17:367. doi: 10.1186/s12974-020-02038-2 (PMC7709339; doi:10.1186/s12974-020-02038-2)

## Slide 1
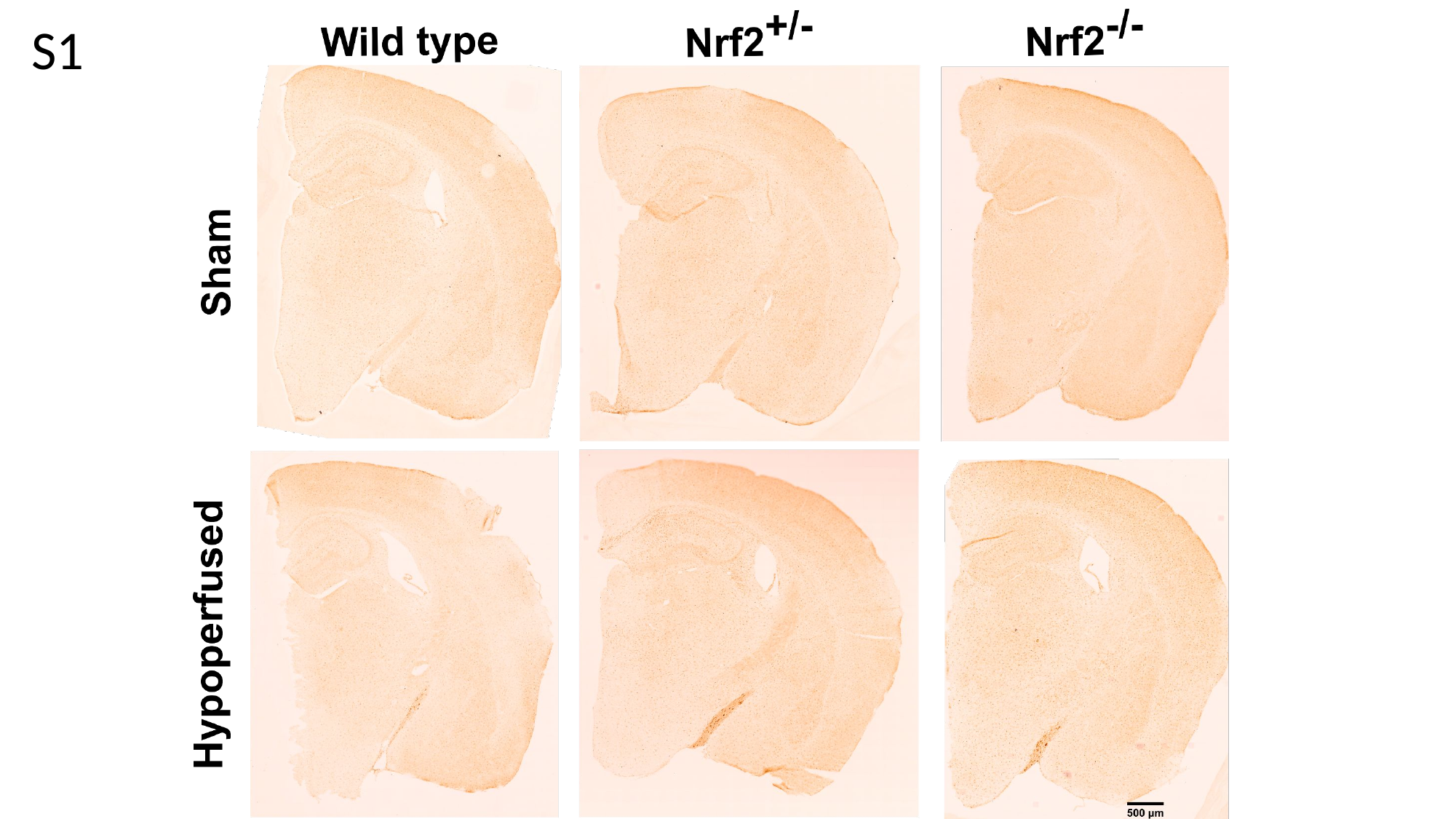

S1

## Slide 2
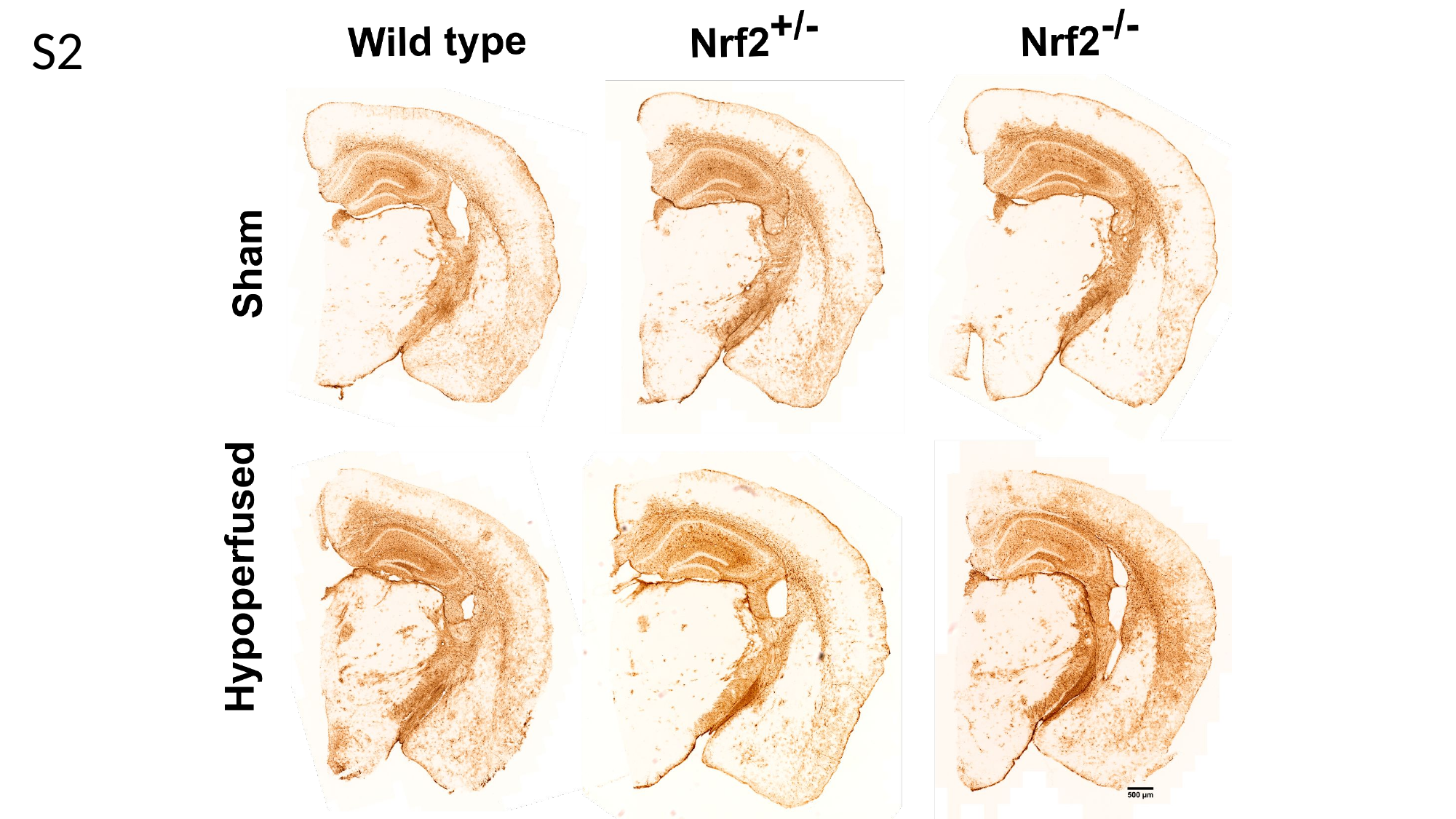

S2

Supplement: Supplementary file 1 — Additional file 1. Supplemental figures [file 12974_2020_2038_MOESM1_ESM.pptx]
